# An Enhanced Offset Tracking Method: Providing Auxiliary Information for DInSAR Phase Filtering in Urban Areas

**DOI:** 10.3390/s23083802

**Published:** 2023-04-07

**Authors:** Qingyu Liu, Xiaoqi Lv, Pingping Huang, Wei Xu

**Affiliations:** Key Laboratory of Radar Technology and Application, School of Information Engineering, Inner Mongolia University of Technology, Hohhot 010051, China

**Keywords:** urban remote sensing, DInSAR, offset tracking, Goldstein filtering

## Abstract

In the application of synthetic aperture radar differential interferometry in urban environments, it is easy to regard the phase change in the deformation band of buildings under construction as noise that requires filtering. This introduces an error into the surrounding area while over-filtering, resulting in an error in the magnitude of the deformation measurement results for the entire region and the loss of deformation details in the surrounding area. Based on the traditional DInSAR workflow, this study added a deformation magnitude identification step, determined the deformation magnitude by using enhanced offset tracking technology, supplemented the filtering quality map and removed the construction areas that affect the interferometry in the filtering stage. The enhanced offset tracking technique adjusted the ratio of contrast saliency and coherence via the contrast consistency peak in the radar intensity image, which was used as the basis for adjusting the adaptive window size. The method proposed in this paper was evaluated in an experiment on a stable region using simulated data and in an experiment on a large deformation region using Sentinel-1 data. The experimental results show that the enhanced method has a better anti-noise ability than the traditional method, and the accuracy rate is improved by about 12%. The supplemented quality map can effectively remove the large deformation area to prevent over-filtering while ensuring the filtering quality, and it can achieve better filtering results.

## 1. Introduction

Differential interferometric synthetic aperture radar (DInSAR) is widely used in seismic displacement measurement [1], glacier movement [2], mining surface deformation [3] and other fields due to its high precision, low cost and wide coverage. Changes in the deformation, noise and scattering characteristics of ground objects affect phase feature information. The Goldstein adaptive filtering algorithm, which is widely used in interference image processing, often uses phase features, such as coherence, pseudo-coherence and the phase gradient, to measure the intensity of noise [4,5,6] as the basis for adaptive coefficient adjustment. When the local object type changes or a large deformation occurs, the phase characteristics decrease sharply because of the change in the scattering characteristics of the ground objects. In addition, changes in inclination also affect coherence; therefore, phase characteristics are also used as the basis for determining large deformation areas. When regional deformation factors are more complex than factors above the study area, such as an urban area, there are a large number of areas, such as building construction areas, that change neither the type nor the inclination of ground objects [7,8,9]. The coherence value of these areas is still maintained at a high level, which leads to the traditional filtering algorithm regarding it as noise that requires filtering. While filtering, the error is passed to the surrounding area.

Offset tracking (OT) technology with a theoretical resolution of 1/20 to 1/30 of the pixel resolution is often used for large-scale deformation measurements. For areas where large deformations occur, such as mining areas and glaciers, offset tracking technology and differential interferometry (OT-DInSAR) technology are often combined for measurements [10,11,12,13,14]. However, for urban areas with complex deformation causes, a lot of human power and material resources are required to obtain a large number of deformation areas by means of field investigations [15]. Traditional adaptive window offset tracking technology pays too much attention to the characteristics that cause the measurement accuracy to be susceptible to noise, and it cannot effectively provide reliable deformation magnitude data for DInSAR processing [16].

Many SOTA techniques use deep learning or frequency domain filtering to try to remove the speckle noise that affects the offset tracking results, and they have achieved good results [17,18,19], but it takes a lot of time to remove the noise in a large area. Multiple-look processing is applied as the simplest speckle noise processing method in this paper. Table 1 compares and analyzes the advantages and disadvantages of these methods.

Aiming to address the problem of the coherence value not being able to effectively separate large deformation areas when synthetic aperture radar interference technology is used in complex areas, such as cities, this paper first improves the traditional offset tracking technology and performs offset tracking according to the adaptive window size determined by the Werner signal-to-noise ratio and coherence value. Then, the offset is combined with a coherence quality map in order to guide the algorithm to distinguish different types of decoherence and to perform targeted Goldstein adaptive filtering. The experimental results show that this method can effectively avoid noise interference, better separate high-deformation areas and prevent errors from spreading to the surrounding area while avoiding over-filtering.

The second section of this paper first elaborates the traditional DINSAR technology processing flow, and then it determines the application mode of the offset tracking technology results on the basis of the process. Then, improvements in traditional offset tracking technology and the method used to integrate the improved results into the filtering algorithm are introduced. In the third section, simulation data are used to verify the working conditions of the enhanced offset tracking method in a stable area under different noise conditions. Sentinel-1 data and the field investigation results are combined to analyze the working conditions of the enhanced offset tracking method in a deformation area. Finally, the differences in the filtering results under different algorithms and threshold settings are compared. In the fourth section, the work of this paper is summarized, and suggestions for future work are given.

## 2. Materials and Methods

### 2.1. Materials

The main data used in this paper are Sentinel-1 satellite data. The Sentinel-1 satellite was launched by ESA and carries C-band SAR. The satellite has a revisit period of 12 days for Earth observations. Because of its good coherence, it is widely used in DInSAR terrain deformation detection research [1,2,20]. Due to its open access characteristics, Sentinel-1 data are often used in combination with other data for change detection, such as vegetation and crop classification [21,22]. This paper combines the interferogram obtained by using the phase information of Sentinel-1 data with the offset obtained by the intensity information, which makes the application of Sentinel-1 satellite data in urban interferometry more complete. These data are mainly used (1) in urban large-area interferometry, as they can allow for an effective identification of the construction area, and (2) in precise measurements of the interference of buildings around the construction area, as they allow for the error caused by the construction of buildings to be effectively avoided.

### 2.2. DInSAR Workflow and Improvement

The workflow of DInSAR technology was established on the basis of traditional algorithms, considering the influence of the terrain, noise, satellite orbit, etc. The workflow in this experiment is based on the two-track DInSAR algorithm. The core of the two-track method only uses a reference digital elevation model (DEM) to simulate the terrain phase, thereby eliminating a scene of radar data. The DEM can also assist in the main and auxiliary image registration, thereby simplifying the experimental materials and improving the experimental efficiency.

In the workflow, the Sentinel satellite images before (main image) and after (secondary image) deformation are first obtained, and multi-view processing is performed to reduce speckle noise; then, the images are registered with the help of DEM. The phase difference in the registered image is obtained via conjugate multiplication to form an interferogram. Then, the coherence is estimated, and the obtained coherence coefficient is used as the basis for calculating a phase quality map and filtered according to the quality map. The phase of the interferogram can only be mode 2π, so when the phase change is greater than 2π, the periodicity of the phase leads to a 2π-fold ambiguity problem in determining the true phase of ground objects. Phase unwrapping is the process of solving this 2π ambiguity. The phase map after unwrapping removes the orbital phase and flat phase by referring to DEM and satellite orbit information. Finally, the final deformation result is obtained via phase terrain conversion with the help of invisible deformation reference points.

**Figure 1:**
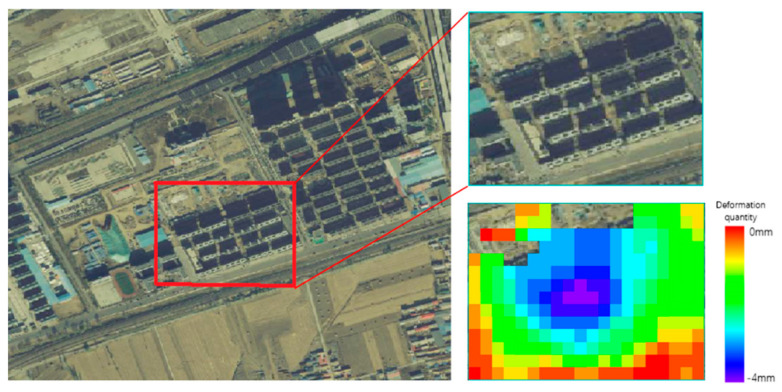
The error results of traditional DInSAR algorithm when applied to a construction area.

In the traditional processing method, the pixels involved in the algorithm are often determined via coherence threshold separation in the filtering stage. However, in the case of urban construction, the coherence of buildings does not sharply decline. Therefore, this information is involved in the filtering algorithm and is considered to be the noise involved in filtering. At the same time, the error information is provided to the phase unwrapping algorithm. In the overall estimation-type unwrapping algorithm, if there is a certain millimeter-scale deformation area around the above area, the entire phase change will be identified as a funnel-shaped deformation area, resulting in a completely wrong deformation result. As shown in Figure 1,the experiment found that this phenomenon is common in cities. For example, in the vicinity of construction areas, there are often road deformations due to transportation.

**Figure 2:**
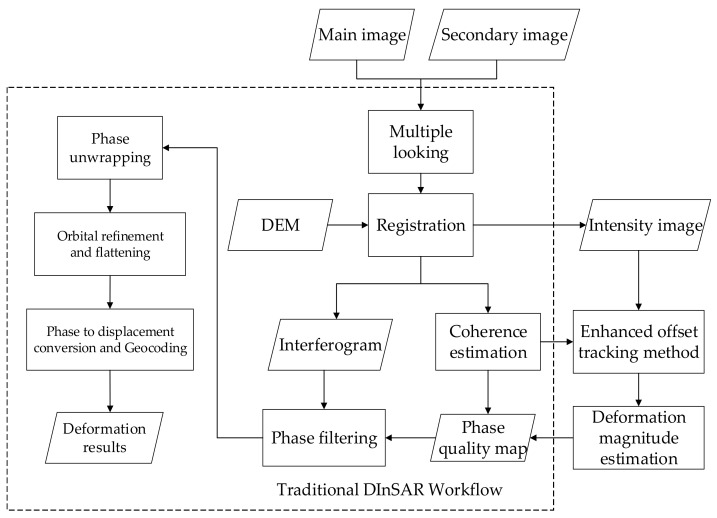
DInSAR filtering algorithm assisted by offset tracking technology.

In order to solve the abovementioned problems, based on the DInSAR workflow, this paper uses a radar intensity image to track the offset, identify the large deformation area, correct the phase quality map and eliminate such errors in the filtering stage. The specific process is shown in Figure 2.

The improved DInSAR workflow registers the intensity images after multi-look processing, and it adopts enhanced offset tracking technology to determine the deformation magnitude in the region. A region with an absolute value of deformation greater than 1 m is determined as the meter-level deformation region, and the corresponding pixel phase quality is set to 0.

### 2.3. Enhanced Offset Tracking Method

Offset tracking technology is mainly divided into that which uses the intensity tracking method and that which uses the coherence tracking method. The coherence tracking method has high requirements for the overall coherence of a region and is not suitable for urban areas with complex coherent environments. Therefore, this paper uses the intensity tracking method to enhance the offset tracking technology. The core idea of this method is to determine the size of the offset by calculating the cross-correlation peak position in the fixed window of the reference image and the search image. The cross-correlation is obtained using Equation (1) [16]:

(1) $$\rho \left(x,y\right)=\left|\frac{\sum \sum \left(f\left(x,y\right)-f_{a}\right)\left(g\left(x+u,y+v\right)-g_{a}\right)}{\sqrt{\sum \sum {\left(f\left(x,y\right)-f_{a}\right)}^{2}\sum \sum {\left(g\left(x+u,y+v\right)-g_{a}\right)}^{2}}}\right|$$

In the equation, (*x, y*) represents the reference image pixel center coordinates; *u* and *v* are the satellite range and the azimuth offset, respectively; *f* and *g* are the pixel intensity of the reference image window and the pixel intensity of the search window, respectively. $f_{a},g_{a}$ are the mean values of the strength of the window.

The selection of the cross-correlation window size directly affects the accuracy of the offset tracking technique. Related studies have shown that [23] the use of a larger cross-correlation window can effectively reduce the impact of noise on the overall results, but this results in different levels of deformation pixels being involved in the calculation, so the results tend to present a mean value of regional deformation. In order to measure the similarity of the same feature in two images, the SNR proposed by Werner [24] is used, and it is the peak value of the correlation coefficient in the cross-correlation window ($\rho_{max}$) divided by the average value of the correlation coefficient other than the 5 × 5 pixels centered on the peak value ($\bar{\rho }$). The SNR is used as the evaluation index to select the adaptive window size.

When the central pixel region is affected by spatiotemporal decoherence, both $\rho_{max}$ and $\bar{\rho }$ are small. As the window expands, $\rho_{max}$ increases to the highest value at a speed higher than that of $\bar{\rho }$. Experiments on specific objects have shown that a low cross-correlation coefficient and high signal-to-noise ratio mismatches occur when the window is small [7,25]. In a large window, the signal-to-noise ratio has a similar value, resulting in the result moving closer to the regional deformation mean. In order to adapt to the characteristics of adaptive parameter changes under large-scale and complex conditions, this paper presents a new evaluation equation:

(2) $$a=\rho_{max}\frac{\rho_{max}}{\bar{\rho }}+\left(1-\rho_{max}\right)\gamma$$

In the equation, $\rho_{max}$ is the cross-correlation coefficient of the cross-correlation peak pixel, $\bar{\rho }$ is the average correlation coefficient of the other pixels in the 3 × 3 window of the cross-correlation peak, $\rho_{max}$***/***$\bar{\rho }$ is the signal-to-noise ratio (SNR) proposed by Werner and γ is the pixel coherence value in the search window [26]. For two radar images, $s_{1}\left(x\right),\;s_{2}\left(x\right)$,

(3) $$\gamma =\frac{\left|\sum s_{1}\left(x\right)*s_{2}{\left(x\right)}^{*}\right|}{\sqrt{\sum {\left|s_{1}\left(x\right)\right|}^{2}*\sum {\left|s_{2}\left(x\right)\right|}^{2}}}$$

**Figure 3:**
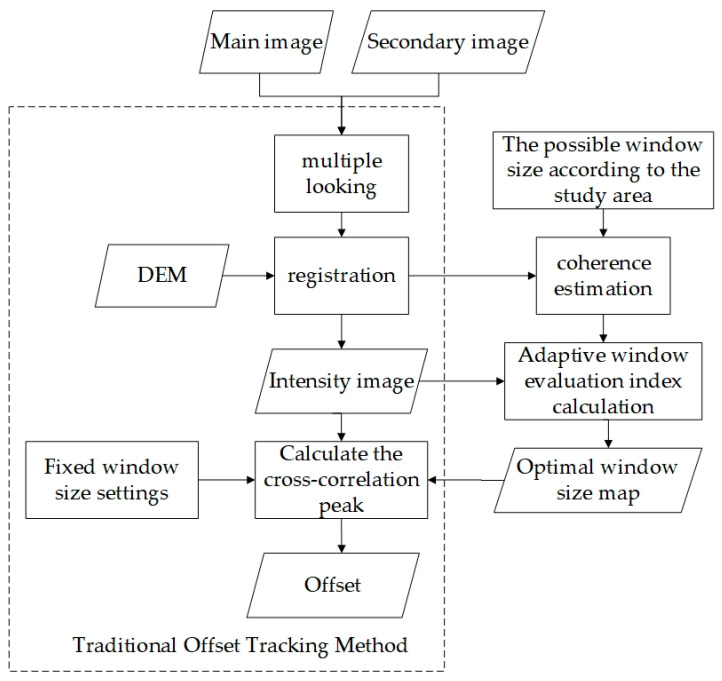
Enhanced and traditional offset tracking technology process comparison diagram.

The enhanced offset tracking technology workflow is shown in Figure 3. Combined with the field investigation, it is considered that, when Sentinel-1 satellite data are selected to track the offset of a city, the possible window size is 3–17.

**Figure 4:**
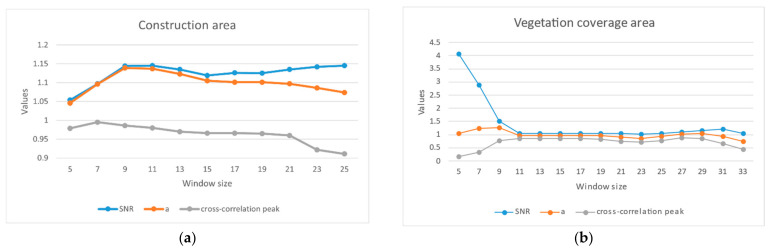
Window size—variation in adaptive coefficient in specific terrain area (**a**); building construction area; (**b**) vegetation coverage area.

In this paper, the correlation $\rho_{max}$ is used as the confidence of the signal-to-noise ratio. When the correlation of the intensity image is poor, the coherence of the complex image is selected as the adaptive index to ensure that the image in the window always maintains a certain consistency before and after processing in order to prevent the occurrence of completely wrong matching. Experiments on specific ground objects show that, when constructing areas, such as those with buildings and viaducts, the offset tracking technology can find high-matching pixels within a certain range, or the window coverage area still has a high contrast. As the window expands, $\rho_{max}$ can be maintained at a high value. The algorithm mainly adjusts the window size using $\rho_{max}$ and the SNR. The index in this paper makes the change in $\rho_{max}$ during the process of window expansion have a more significant impact on the adaptive coefficient, avoiding a situation where the SNR has a similar value when the window is large. The window size is mainly determined by the coherence value, which encourages the window to expand in order to include high-coherence targets. At this time, the window size takes into account both the SNR and the regional coherence. When the construction area is much smaller than the adaptive window, the pixels in the window still have a large average coherence value, and the algorithm can avoid window expansion due to the decrease in the cross-correlation coefficient. In summary, the indicators proposed in this paper can improve the anti-noise performance of the algorithm while preventing the excessive expansion of the window from affecting the identification of small deformation areas. Figure 4 shows the variation characteristics of different adaptive parameters in the face of characteristic features. It can be seen that the adaptive parameters proposed in this paper can effectively avoid the problem of the ***SNR*** approaching the regional deformation mean in the presence of a large window and mismatching.

### 2.4. Adaptive Goldstein Filtering Algorithm

Goldstein filtering is an interferogram filtering algorithm proposed by Goldstein and Werner. The core idea of Goldstein filtering is to divide an interferogram into several small overlapped windows for frequency domain filtering [5]; for the image Z(*u,v*) to be filtered, the filtered image H(*u,v*) must satisfy

(4) $$H\left(u,v\right)=S{\left\{\left|Z\left(u,v\right)\right|\right\}}^{\alpha }Z\left(u,v\right)$$

*S* is the smoothing operator, and α is the filtering parameter.

Coherence can reflect the level of noise to a certain extent. A method proposed by Baran is employed to adaptively determine α by using coherence [27]:

(5) $$\alpha =1-\bar{\gamma }$$

In the equation, $\bar{\gamma }$ is the average coherence value. This method equates all decoherence cases with noise for adaptive filtering, and the occurrence of decoherence is related to both deformation and noise. Therefore, it also over-filters while suppressing noise and losing deformation details.

It is found that, in an urban environment, the coherence of ground objects with good scattering characteristics, such as buildings and roads, will not decrease sharply when they are constructed (in the experiment, the mean value of coherence in the construction area of a building area changes from 0.9 to 0.62, and the road area changes from 0.8 to 0.55). A filter with a coherence quality map as the adaptive index will filter out the decoherence caused by construction as noise. Losing the deformation characteristics of the area will affect the coherence mean value of other nearby ground objects and spread the error to the surroundings.

**Figure 5:**
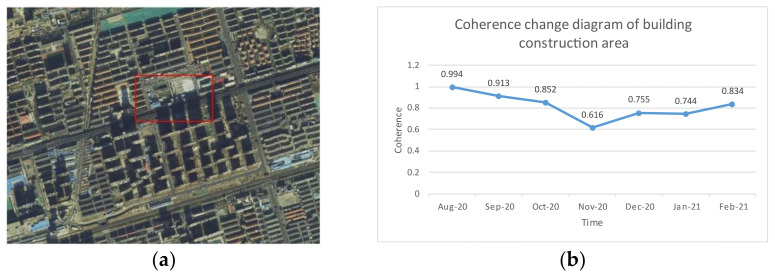
Building construction area overview and coherence changes: (**a**) overview of building construction area; (**b**) coherence change diagram of building construction area.

**Figure 6:**
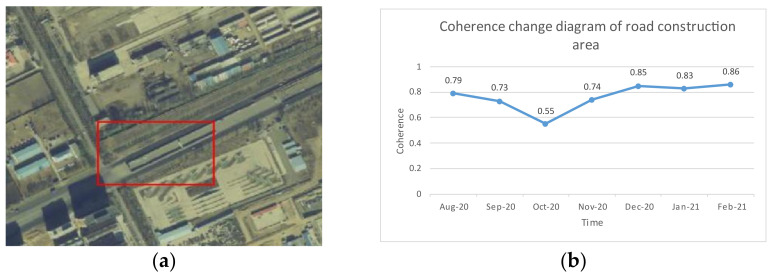
Road construction area overview and coherence changes: (**a**) overview of road construction area; (**b**) coherence change diagram of road construction area.

Figure 5 and Figure 6 show the coherence changes in an urban construction area and a road construction area before, during and after construction. Figure 5 shows that, in the early stage of building construction, the construction of temporary buildings is mainly carried out, and the coherence decreases slowly. Coherence decreases significantly during ground excavation. After the underground work is completed, the coherence gradually increases after the ground is backfilled, and the coherence remains at a general level during the construction phase of the building ground. Figure 6 shows that the coherence of a viaduct decreases significantly in the bridge foundation construction stage. After the completion of the bridge foundation construction, the coherence is restored to a level similar to that of the original road. As the bridge is built, the coherence is improved due to the change in the ground material. The above situation makes DInSAR technology, PSInSAR technology and SBAS-InSAR technology regard it as nonlinear deformation or noise, thus affecting the reliability of the final result.

In engineering practice, in order to effectively deal with an area where the radar echo is weak or the ground object category changes, the coherence threshold involved in filtering is often set. For pixels with particularly low coherence values, they are not involved in the overall filtering algorithm in order to avoid the introduction of errors into other areas. In this paper, combined with the proposed enhanced offset tracking method and engineering practice, the low-coherence areas outside of the measurement range of interference technology and offset tracking technology, such as roads frequently passed by construction vehicles, water bodies and dunes with a weak radar reflection, are excluded due to the coherence threshold. In construction areas, road construction areas and other areas where meter-level deformation occurs, according to the offset tracking results, the corresponding pixel coherence quality is set to 0. The coherence quality value of the excluded region is not involved in the calculation of γ. Considering that the enhanced offset tracking method still results in some erroneous results and that the noise causing the wrong results is sporadic and independent, a 3 × 3 window is selected to judge and filter the quality threshold of the region. It is considered that the point where the average quality of the window is lower than the threshold is not a millimeter-level deformation, and it is not involved in the calculation of adaptive filtering weights.

## 3. Experiment and Analysis

### 3.1. Experimental Materials

In this paper, Sentinel-1 satellite data on 30 August 2021 and 10 November 2021 are selected as experimental materials to determine large-scale urban deformation. The open access software SNAP is used to process the above data, such as orbit correction, registration and fixed window offset tracking. Version 3.10 Python programming simulation experiments were used. Reference optical images selected in the field trip were obtained from the Sentinel-2 satellite and Rivermap.

### 3.2. Enhanced Offset Tracking Method

Limited by the workload of field research, this paper divides the experiment of improving offset tracking technology into two parts. The first part is composed of simulated data experiments, which aim to compare and verify the work of different algorithms in the face of different levels of noise when there is no large deformation area. In the second part, an urban area containing buildings and viaduct construction is selected to track the offset of the collected radar images, and a field survey is carried out for a comparative analysis.

#### 3.2.1. Simulation Data Experiment

The system noise model of the SAR image is the multiplicative noise model [28,29]:

(6) $$I\left[m,n\right]=S_{t,\theta }\left[m,n\right]*w\left[m,n\right]$$

where $w\left[m,n\right]$ is the Gamma noise, and $S_{t,\theta }\left[m,n\right]$ is the noiseless SAR signal, which is related to the acquisition time and the radar line of sight angle. $I\left[m,n\right]$ is the SAR signal obtained in practice. The probability density of the radar image after multiple-look processing with multiple-look number *L* is as follows:

(7) $$pdf\left(w\right)=\frac{L^{L}w^{L-1}}{\Gamma \left(L\right)}e^{-Lw}$$

where $\Gamma \left(L\right)$ is the factorial of (L−1).

Obviously, the radar intensity image noise is multiplicative noise, and the phase image noise is additive noise. In view of the above model, this paper uses actual measurement data containing various urban features as assumed noise-free samples to add noise twice in order to conduct simulated data experiments, and the experiments are carried out on the noise of different views. The simulated data size is 191 × 358, the mean intensity is 1.58 and the standard deviation is 41.58. The data include urban and rural buildings, urban and rural roads, water bodies, sand dunes and other features. The experimental hypothesis area is completely without any shape, and it is only affected by noise. The offset tracking technology uses 20-times oversampling, measured in the radar line of sight, to produce more than one-twentieth of pixel (excluding) deformation as an error result.

**Figure 7:**
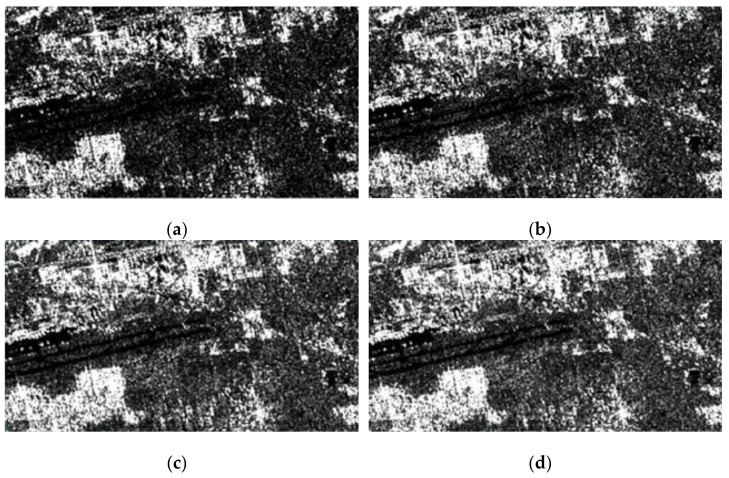
Experimental samples under noise of different multiple-look numbers. (**a**) Main image when L = 3; (**b**) secondary image when L = 3; (**c**) main image when L = 7; (**d**) secondary image when L = 7.

**Figure 8:**
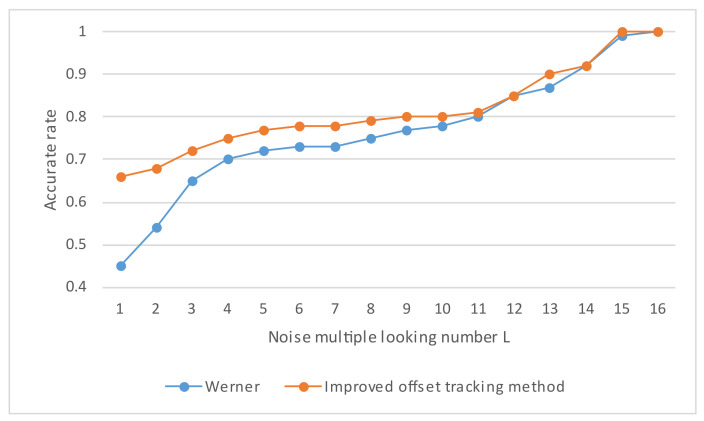
The correct rate of samples with different multiple-look numbers.

Figure 7 is a sample of different multiple-look numbers. Due to the randomness of noise, this paper conducts multiple experiments to obtain the average value, and the correct rate is shown in Figure 8.

In the simulation data, it can be seen that, in the window with the SNR as the adaptive coefficient, in the case of fewer multiple-look numbers, the correct rate of multiplicative noise is much lower than that of the adaptive coefficient obtained using the method proposed in this paper, and the correct rates of the two coefficients gradually become closer with an increase in the multiple-look number. When the multiple-look number is low, the image is seriously affected by multiplicative noise, and the distribution of the high-coherence regions is scattered. The situation of a low cross-correlation peak and a high signal-to-noise ratio is significant when the SNR is the adjustment index of the adaptive coefficient. The method of simply pursuing a high signal-to-noise ratio and ignoring the correctness of matching leads to a high error rate in regional results. The difference between the two adaptive coefficients can reach 20% to 15%. With an increase in the multiple-look number, the influence of noise is gradually weakened. When the number of views is more than 4, the correct rates of both sides gradually become closer. When the multiple-look number is 4 to 7, the accuracy of this paper is better than the SNR in any sample experiment that is the same, with an accuracy that is 2% to 5% higher. When the number is greater than 7, a very small number of samples have an equal level of accuracy, and most of the samples have an accuracy higher than the SNR by about 2% to 5%. As the multiple-look number rises above 10, there is no longer a significant difference between the two indicators. In summary, the adaptive index is more resistant to multiplicative noise.

It should be noted that the choice of window size only determines whether the offset slips to the regional average offset, so the experimental results of simulated deformation region data will not be too different from those of the above experiments.

#### 3.2.2. Real Data Experiment

**Figure 9:**
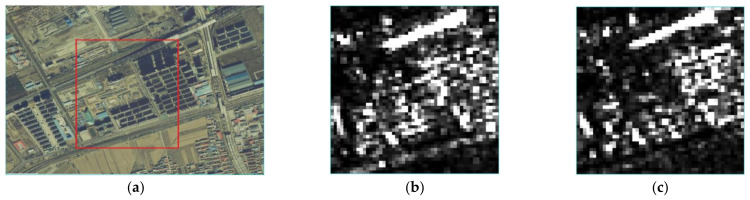
The intensity map and coherence map of main and auxiliary images: (**a**) research area (red box) and surrounding overview. (**b**) Main image (collected on 30 August 2021); (**c**) secondary image (collected on 10 November 2021).

**Figure 10:**
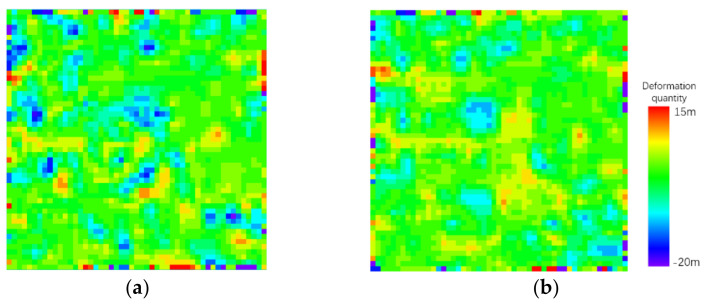
Results of offset tracking technology: (**a**) conventional method; (**b**) method proposed in this article.

In this paper, the results of different methods are compared and analyzed by conducting experiments on small areas where building construction and road construction are being carried out. In this paper, a construction area in the Jinchuan Development Zone of Hohhot City is selected as the experimental area, and Sentinel-1 satellite data are used for experiments. The experimental area is 51 pixels× 51 pixels (about 1 square kilometer). The main and secondary intensity images and an area overview are shown in Figure 9. The construction area where the field investigation is carried out is marked with a red box in the coherence coefficient diagram. The offset tracking results obtained using the two methods are shown in Figure 10.

**Figure 11:**
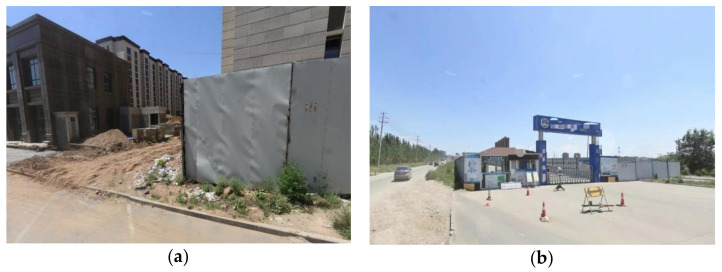
Examples of construction area boundary inspection: (**a**) road construction boundary example; (**b**) building construction boundary example.

**Figure 12:**
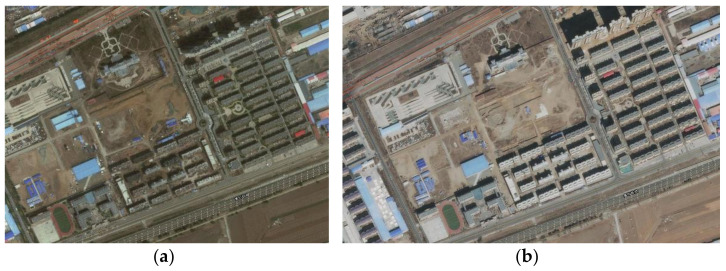
Optical remote sensing images of the corresponding period: building area before and after construction; (**a**) main image; (**b**) secondary image.

In this experiment, the optical image is first geocoded and then registered with the radar image, followed by a comparative study. Combined with the field investigation results and historical optical remote sensing satellite images, the deformation of the study area is determined. The field investigation mainly delineates the type and boundary of the regional inland objects, judges whether the regional inland objects are in a continuous construction state, investigates whether the deformation reaches the meter level and determines the construction boundary. Examples of the boundary conditions and optical remote sensing images are shown in Figure 11 and Figure 12.

The road construction area belongs to the connection between the end of the Jinhai Viaduct and an ordinary road. If the pixels of the road area meet one of the following conditions in the field investigation, it is determined that meter-level deformation occurred:

1. The road section is located in the construction area at the time of the inspection and is in a state of continuous construction, and the viaduct erection is completed.
2. The road belongs to the construction area at the time of the investigation, and it is not in a continuous construction state at the time of the field investigation, but the viaduct was erected during the first and last field investigations.
3. The optical remote sensing image shows that the erection of the viaduct is completed.

In the field investigation, no meter-level deformation is found in the non-construction area of the road.

Considering the limitations of ground investigations and optical remote sensing satellite images, if one of the following conditions exists in the pixels of the building area in the field investigation, it is determined that meter-level deformation occurred:

1. Non-temporary buildings are in a state of continuous construction.
2. Temporary buildings have been newly built or demolished.
3. The building materials or facilities stacked in the construction area have disappeared or moved.
4. The optical remote sensing images show significant changes in regional architecture.

**Figure 13:**
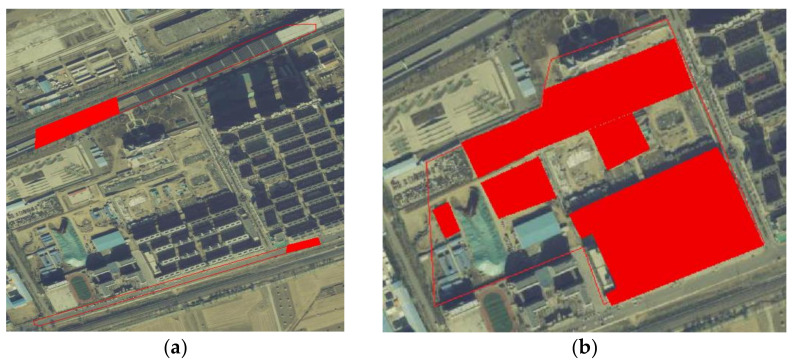
Area where deformation reaches meter level: (**a**) road construction area; (**b**) building construction area.

Because there are many meter-level deformation areas in the construction area, the construction area is first divided and then carefully investigated. Combined with the optical remote sensing image taken near the sampling time point of the main and secondary images, the field investigation is carried out according to the pixels in the area, and the pixels in the meter-level deformation area are determined according to whether the maximum deformation exceeds one meter, as shown in Figure 13. The areas involved in the investigation are marked with red boxes. The deformation areas are shown in red in the figure. According to the results of the field investigation, the accuracy of the recognition for the meter-level deformation zone of different algorithms can be obtained, as shown in Table 2 and Table 3. In the table, it is considered that areas where the offset is above 1 m are deformation areas; otherwise, they are non-deformation areas. The situation in which a deformation area in the field investigation is regarded as a non-deformation area is considered to produce the wrong judgment number of deformation areas. The case in which other regions are regarded as deformation regions produces the wrong number of non-deformation regions. The ratio of the correct number of pixels to all pixels in the whole region is the correct rate.

It can be seen in the above table that the correct numbers of the two adaptive indicators in the deformation region are similar, and the main difference comes from a misjudgment of the non-deformation region. The window with the SNR as the adaptive index is prone to misjudgment, and the anti-noise performance is significantly lower than that of the method proposed in this paper. The algorithm proposed in this paper can ensure the effective identification of the deformed region while reducing the number of misjudgments in the non-deformed region.

The lower right of the experimental area is grassland. The algorithm with the SNR as the adaptive index considers the uplift of 7–10 m in this area to be the result of mismatching. The adaptive algorithm with the SNR as the adaptive index considers some construction buildings to have a settlement of about 5–10 m. The above two cases are the result of an error match when selecting a small window. The algorithm in this paper avoids the occurrence of false matching, which shows that it can effectively suppress the influence of vegetation on the intensity information of ground objects, can prevent the occurrence of false matching in construction areas and has better reliability.

### 3.3. Auxiliary Goldstein Filtering Algorithm

In this paper, the differences between the proposed algorithm and the Baran filtering algorithm are compared by filtering the interferogram of the above experimental area. The denoising ability of the algorithm is measured using the number of residual points, and over-filtering is measured using the cross-correlation coefficient of the noise and the filtered phase [30].

**Figure 14:**
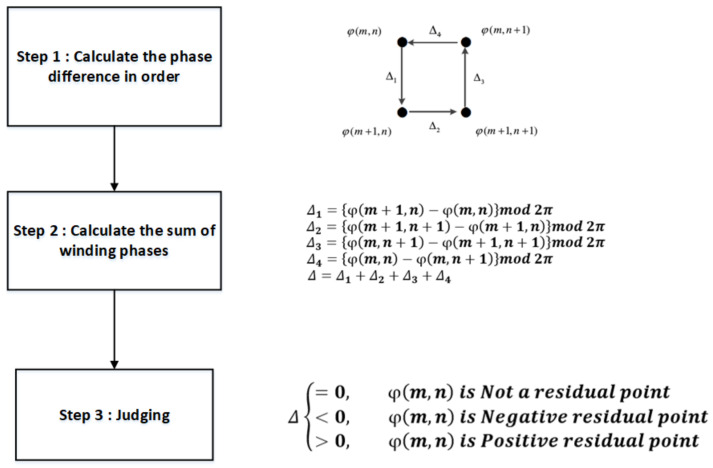
Residual point determination flowchart.

A residual point is a point with a discontinuous phase in an interferogram. The cause of this may be noise and a large deformation. In DInSAR processing, residual points affect the accuracy of phase unwrapping. Because the number of residual points caused by a large deformation is small, the number of residual points can be used to measure the noise removal ability of the filtering algorithm. For a point $\varphi \left(m,n\right)$ in the interferogram, the process of determining the residual point consists of three steps, as shown in Figure 14.

The phase noise in the interferometric phase model is additive noise, and the noise phase is independent of the true phase. Therefore, the cross-correlation coefficient of the phase difference before and after filtering ($\varphi_{d}$) and the filtered phase ($\varphi_{f}$) can be used to measure the over-filtering of the filtering algorithm. For a window of size M × N, the technique of the cross-correlation coefficient (CCC) is shown in Equation (8):

(8) $$CCC=\frac{\sum_{i=0}^{M-1}\sum_{j=0}^{N-1}\left(\varphi_{f}\left(i,j\right)-\bar{\varphi_{f}}\right)\left(\varphi_{d}\left(i,j\right)-\bar{\varphi_{d}}\right)}{\sqrt{\sum_{i=0}^{M-1}\sum_{j=0}^{N-1}{\left(\varphi_{f}\left(i,j\right)-\bar{\varphi_{f}}\right)}^{2}}\sqrt{\sum_{i=0}^{M-1}\sum_{j=0}^{N-1}{\left(\varphi_{d}\left(i,j\right)-\bar{\varphi_{d}}\right)}^{2}}}$$

**Figure 15:**
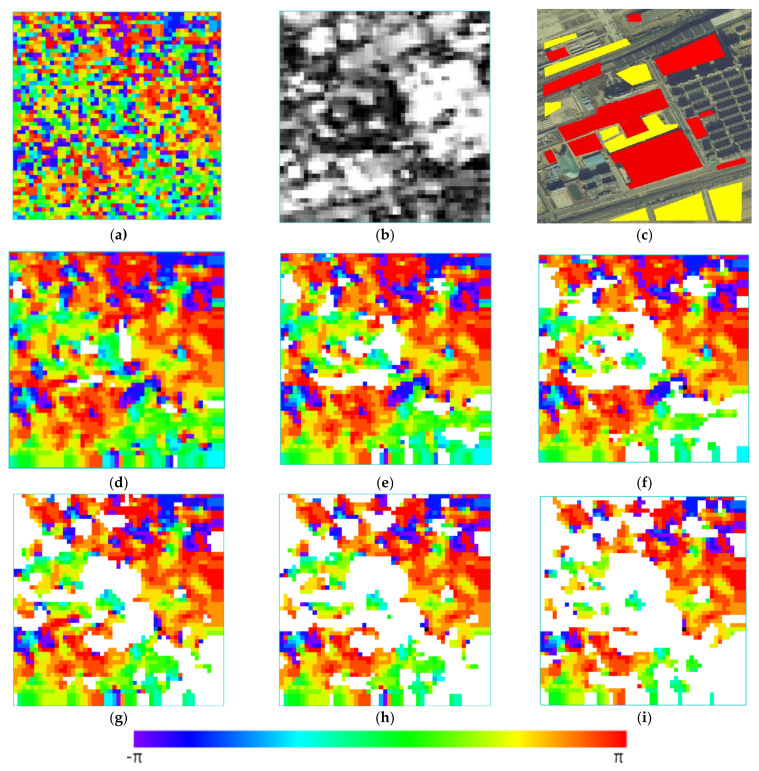
Filtering results and comparative analysis: (**a**) unfiltered phase map; (**b**) coherence map; (**c**) optical remote sensing image and deformation magnitude classification; (**d**) Baran filtering method, threshold 0.2; (**e**) Baran filtering method, threshold 0.3; (**f**) Baran filtering method, threshold 0.4; (**g**) improved filtering method, threshold 0.2; (**h**) improved filtering method, threshold 0.3; (**i**) improved filtering method, threshold 0.4.

A subjective analysis of the filtering effect is carried out by combining the field investigations. In an interferogram, the fewer the residual points, the stronger the denoising ability of the filtering algorithm. The smaller the cross-correlation coefficient between the noise and the filtered phase, the stronger the detail retention ability and the less the over-filtering. The regional coherence coefficient is shown in Figure 15b. The higher the gray value, the closer the coherence to 1.

The boundary of ground objects is defined by a field investigation, and the ground objects are divided into a millimeter-level deformation zone, a meter-level deformation zone and other deformation zones according to the type and reality of the ground objects. The meter-level deformation zone mainly includes buildings and roads under construction; the other deformation zones mainly include pasture land, roads in the construction area and township roads frequently passed by construction vehicles. The millimeter-level deformation zone mainly includes buildings, roads, railways and less-used township roads. As shown in Figure 15c, in the optical images, the meter-scale deformation area is marked in red, and the other deformation areas are marked in yellow.

The experimental results are shown in Figure 15d–f. The statistical characteristics of the filtered phase are shown in Table 4.

It can be seen in Figure 15 that the traditional algorithm has a poor recognition ability for buildings and roads in construction areas. The red area in the lower-right corner of Figure 15c is always regarded as a millimeter-scale deformation area. In the field investigation, this part of the building completed the construction, adding at least one floor. The algorithm in this paper can ensure the normal deformation monitoring of non-construction buildings while identifying these areas, indicating the improved reliability of the algorithm.

By comparing the results of the Baran algorithm obtained using different thresholds, it can be seen that the pasture fields in the region and the roads in and out of the construction area used by vehicles gradually separate with an increase in the coherence threshold. The algorithm reduces the number of residual points without filtering. This shows that the separation of non-millimeter-scale deformation regions can eliminate residual points while preventing the adaptive algorithm from over-filtering the surrounding area as noise. When the coherence reaches a threshold of 0.4, the above regions are basically separated, and the coherence threshold is further increased to 0.5, resulting in the exclusion of some unconstructed roads and a decrease in the algorithm’s coverage.

Using the same threshold settings, the cross-correlation coefficient of the millimeter-level deformation pixel noise is significantly reduced, and over-filtering is reduced. The residual points remain consistent or decrease slightly. Combined with the coherence coefficient diagram, it can be seen that the phase change in a large number of deformations caused by construction is stable, and few residual points are generated. However, the frequency domain adaptive filtering of these regions will result in the wrong results, which will also lead to an increase in the noise cross-correlation coefficient and affect the overall quality of the algorithm. It can be found from the comprehensive field investigation that the algorithm in this paper effectively removes the roads and buildings in construction areas that cannot be excluded by the coherence threshold. The algorithm can achieve good filtering results in a low-coherence environment, and it can improve the overall reliability while ensuring that more millimeter-level deformed pixels are involved in the calculation.

## 4. Limitations and Future Work

1. Insufficient application of multi-source SAR data

In the improved offset tracking algorithm, due to the resolution of the Sentinel-1 satellite, the accuracy of offset tracking technology can only reach 1 m. Areas with a severe deformation of −1 m to 1 m may be regarded as millimeter-scale deformation areas. Using higher-resolution SAR data can effectively solve this problem, but the registration of different SAR data and the fusion of different resolution data may result in new challenges in future work.

2. Partial deformation zone simulation data are difficult to generate

Among the many studies on offset tracking technology, often, only real data are used for verification, and only some studies using intensity information use simulated data for experiments. It is difficult to generate simulated data because the phase of deformed pixels is difficult to simulate. In these simulation data experiments for offset tracking technology, funnel-shaped deformation is often targeted, so the interpolation method cannot be used to generate simulation data. This requires more research in future work.

## 5. Conclusions

This paper proposes an enhanced offset tracking method to avoid the influence of building construction on synthetic aperture radar interferometry. Compared with the traditional method, the accuracy of the proposed method is improved by 12%. Based on this method, the traditional DInSAR technology has improved ability to remove these large deformation regions in the filtering stage. Experiments showed that the improved DInSAR algorithm process can effectively remove the construction area, reduce over-filtering and ensure the filtering quality.

## Figures and Tables

**Table 1:** The advantages and disadvantages of different techniques.

Techniques	Advantages	Disadvantages
DInSAR	1. Good robustness 2. High precision 3. Strong anti-noise ability 4. Efficient, fast	1. Only millimeter-level deformation can be measured 2. Easily affected by large deformation areas
Offset tracking	1. Good robustness 2. Efficient, fast 3. Low requirement for image coherence	1. Precision is relatively low 2. Poor anti-noise ability 3. Only large deformations can be monitored
OT-DInSAR	1. Deformation of all magnitudes can be measured 2. Strong anti-noise ability	1. Requires more computing resources 2. Only for specific areas
Deep learning reinforcement offset tracking	1. High precision 2. Strong anti-noise ability 3. Low requirement for image coherence	1. Requires more computing resources 2. Only for specific areas

**Table 2:** Different adaptive indices and field investigation in road area.

Measurement Method	Number of Deformed Pixels	Number of Non-Deformed Pixels	Error Number of Deformation Region	Error Number of Non-Deformation Zone	Accuracy Rate
Improved	115	222	7	26	85.5%
Werner	153	184	16	73	73.6%
Site visit	96	241	\	\	\

**Table 3:** Different adaptive indices and field investigation in building area.

Measurement Method	Number of Deformed Pixels	Number of Non-Deformed Pixels	Error Number of Deformation Region	Error Number of Non-Deformation Zone	Accuracy Rate
Improved	517	249	70	77	80.8%
Werner	626	140	74	190	65.6%
Site visit	510	263	\	\	\

**Table 4:** Filtering effect using different algorithms and threshold settings.

Algorithm (Threshold)	Residual Points	CCC	Millimeter Deformation Pixel
Unfiltered phase map	475	/	1604
Baran (0.2)	90	0.698	2523
Baran (0.3)	78	0.696	2277
Baran (0.4)	58	0.696	1916
Improved (0.2)	90	0.688	1831
Improved (0.3)	75	0.686	1587
Improved (0.4)	53	0.683	1299

## Data Availability

Not applicable.
